# Electrospun Bis(2,4,4-trimethylpentyl) Tetradecyltrihexylphosphinate/Polyacrylonitrile Nanofiber Membranes with Enhanced Benzene, Toluene, and Xylene Adsorption Performance and Regenerability

**DOI:** 10.3390/nano15100711

**Published:** 2025-05-09

**Authors:** Changchang Wang, Xinrong Tao, Fengjen Chu

**Affiliations:** 1 School of Safety Science and Engineering, Anhui University of Science and Technology, No. 168, Taifeng St., Huainan 232001, China; ccwang145@163.com; 2 School of Public Health, Anhui University of Science and Technology, No. 15, Fengxia Rd., Hefei 231131, China; xrtao1116@hotmail.com

**Keywords:** volatile organic compounds, [P_(14)666_]TMPP/PAN, electrospinning, benzene, toluene, xylene, ionic liquid membrane, adsorption

## Abstract

Volatile organic compounds (VOCs) significantly contribute to atmospheric pollution and present considerable health hazards. This study involves the fabrication of a novel ionic liquid/polymer nanofiber membrane, [P_(14)666_]TMPP/PAN, using electrospinning, and its subsequent evaluation for adsorption performance concerning typical aromatic volatile organic compounds—benzene, toluene, and xylene. The membranes were methodically analysed utilising SEM, TGA, FTIR, and XRD techniques. Adsorption tests indicated that augmenting the [P_(14)666_]TMPP loading improved VOC uptake, with the 80 wt% ionic liquid membrane attaining maximum adsorption capacities of 1466.81, 569.14, and 456.29 mg/g for benzene, toluene, and xylene, respectively—signifying enhancements of 23.6-, 4.8-, and 8.4-fold compared to pristine PAN. Furthermore, regeneration studies validated consistent performance across four adsorption–desorption cycles. The results underscore the efficacy of electrospun [P_(14)666_]TMPP/PAN membranes as reusable adsorbents for the elimination of volatile organic compounds in air purification applications.

## 1. Introduction

Volatile organic compounds (VOCs) constitute a large category of air pollutants characterised by elevated vapour pressure and low boiling temperatures, playing a crucial role in the generation of PM_2.5_ and ground-level ozone. Their chemical interactions with air compounds like SO_2_ and NO_x_ facilitate secondary pollution, endangering both environmental integrity and human health [1,2,3,4,5,6]. Aromatic chemicals such as benzene, toluene, and xylene (BTX) are notably perilous among VOCs because to their persistence, potential for bioaccumulation, and toxicity; prolonged exposure has been associated with oxidative stress, hepatic damage, and haematologic diseases [7].

Diverse techniques for VOC removal have been investigated, including condensation [8], combustion [9], catalytic oxidation [10], membrane separation [11], and biological treatment [12]. Among these methods, adsorption has garnered the greatest interest owing to its simplicity, cost-effectiveness, and high efficiency [13,14]. Traditional adsorbents, including activated carbon and molecular sieves, frequently exhibit drawbacks such as inadequate selectivity, elevated regeneration costs, and restricted recyclability, consequently constraining their practical utility.

Ionic liquids (ILs), consisting of organic cations and anions, have arisen as viable alternatives owing to their minimal vapour pressure, substantial thermal and chemical stability, and adjustable physicochemical characteristics [15,16,17]. Ionic liquids exhibit exceptional solubility for both polar and non-polar volatile organic compounds, facilitated by many interaction mechanisms including π–π stacking, C–H···π interactions, and hydrogen bonding. Research indicates that benzene demonstrates the greatest solubility in ionic liquids (ILs) among BTEX compounds, attributable to its robust π-conjugation affinity, and ILs are also proficient at dissolving low-polarity molecules [18,19,20].

Nonetheless, the liquid characteristics of ionic liquids pose difficulties in handling and recovery, necessitating their immobilisation on solid substrates. The integration of ionic liquids into membrane systems has resulted in many formats: supported ionic liquid membranes (SILMs), ionic liquid polymer membranes (ILPMs), and poly (ionic liquid) membranes (PILMs) [21,22,23]. However, numerous instances exhibit leakage, insufficient surface area, or structural instability.

Electrospinning is a flexible and scalable method for producing polymer nanofiber membranes characterised by high porosity, adjustable fibre diameter, and extensive specific surface area [24]. These characteristics render electrospun membranes very appealing for adsorption-based separations. The incorporation of ionic liquids into electrospun matrices is a promising approach to merge the selective selectivity of ionic liquids with the structural and surface benefits of nanofibers. Previous research has shown that IL-modified membranes effectively eliminate VOCs including toluene and methanol, and recent advancements indicate progress in scaling IL-based membranes from laboratory settings to industrial applications [23].

This study presents the construction of an innovative [P_(14)666_]TMPP/PAN composite membrane using electrospinning. A series of membranes with varying IL loading (30–80 wt%) were synthesised and assessed for their adsorption efficacy towards BTX. Morphological, structural, and thermal analyses were conducted to elucidate material features, whereas adsorption and regeneration experiments validated substantial absorption capabilities and stability across numerous cycles. This is the inaugural report employing electrospun IL/PAN membranes for direct BTX adsorption, presenting a novel approach for the advancement of high-performance, recyclable VOC adsorbents.

## 2. Materials and Methods

### 2.1. Materials

Polyacrylonitrile (PAN, Mw = 8000 g/mol) was acquired from Shanghai Macklin Biochemical Co., Ltd. (Shanghai, China). N,N-dimethylformamide (DMF, 99%, analytical reagent grade), utilised as the solvent, was provided by Shanghai Titan Technology Co., Ltd. (Shanghai, China). The ionic liquid bis(2,4,4-trimethylpentyl) tetradecyltrihexylphosphinate, referred to as [P_(14)666_]TMPP, together with the volatile organic chemicals—benzene, toluene, and xylene (BTX, 99%, AR)—were procured from Shanghai McLean Biochemical Co., Ltd. (Shanghai, China). All compounds were utilised without additional purification.

### 2.2. Preparation of [P_(14)666_]TMPP/PAN Composite Membrane via Electrospinning

The [P_(14)666_]TMPP/PAN spinning solution was prepared by dissolving 0.7 g of PAN in 5 g of DMF while maintaining continuous stirring at ambient temperature for 6 h. Subsequently, 0.3 g of [P_(14)666_]TMPP was incorporated, and the mixture was agitated for 12 h to achieve homogeneity, resulting in a solution with 30 wt% ionic liquid concentration. Additional formulations with varying [P_(14)666_]TMPP mass ratios were created using the same process, as outlined in Table 1.

The electrospinning procedure was conducted by placing the prepared solution into a syringe equipped with a stainless steel needle of 0.3 mm inner diameter. A syringe was affixed to a programmable infusion pump, and a voltage of 20 kV was placed between the needle and a grounded collector located 10 cm distant. The solution was administered at a flow rate of 1.2 mL/h, and the nanofibers were gathered on a spinning drum functioning at 200 rpm. In the course of electrospinning, DMF swiftly evaporated, leading to the deposition of solid fibres. The gathered membranes were then dried in an oven at 60 °C for 12 h to eliminate residual solvent and moisture.

**Figure 1:**
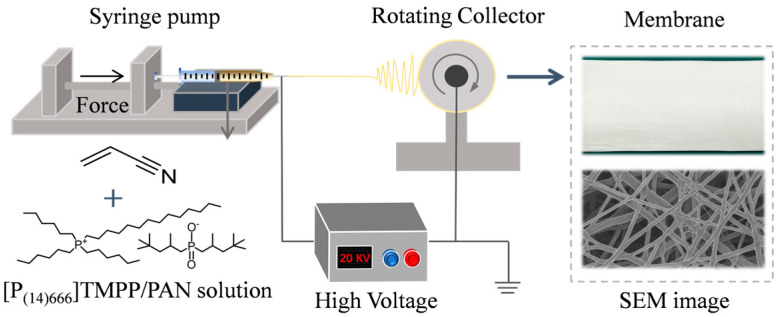
Schematic illustration of the electrospinning.

A series of nanofiber membranes containing ionic liquid (IL) concentrations of 0, 30, 40, 50, 60, 70, and 80 wt% were synthesised, labelled as PAN, 30 TMPP/P, 40 TMPP/P, 50 TMPP/P, 60 TMPP/P, 70 TMPP/P, and 80 TMPP/P, respectively. Figure 1 depicts the configuration for electrospinning.

### 2.3. Characterisation

The structural, thermal, and morphological characteristics of PAN, [P_(14)666_]TMPP, and TMPP/P composite membranes were methodically analysed. X-ray diffraction (XRD) analysis was performed utilising a BRUKER D8 ADVANCE diffractometer (Ettlingen, Germany) with Cu Kα radiation across a 2θ range of 5–80° and a scanning duration of 5 min per sample to assess crystalline structures.

The Fourier transform infrared spectroscopy (FTIR, Nicolet iS20, Waltham, MA, USA) was utilised to examine functional groupings. To mitigate moisture interference, [P_(14)666_]TMPP was pre-dried at 120 °C for 2 h, and the 50 TMPP/P membrane was sectioned into 1 cm × 1 cm pieces. The spectra were obtained within the range of 4000–400 cm^−1^ at a resolution of 4 cm^−1^.

Thermal stability was evaluated using thermogravimetric analysis (TGA 2) in a nitrogen environment. Samples were subjected to heating from 30 °C to 800 °C at a rate of 10 °C/min, accompanied by a nitrogen flow of 20 mL/min.

The fibre morphology of the PAN and TMPP/P membranes was examined using scanning electron microscopy (SEM, HITACHI Regulus 8100, Tokyo, Japan) at an accelerating voltage of 3 kV. The distribution of fibre diameters was assessed using Nano Measurer software (1.2). To ensure statistical validity, 40 individual fibres were systematically measured in each SEM picture from top to bottom and left to right, and the average fibre diameter was subsequently computed.

### 2.4. VOCs Adsorption Experiment

The adsorption efficacy of the membranes for volatile organic chemicals (BTX: benzene, toluene, and xylene) was assessed utilising a bespoke static vapour-phase adsorption apparatus [25,26,27]. The methodology employed was as follows:

(1) Sample Preparation: Each membrane specimen was sectioned into dimensions of approximately 3 cm × 5 cm, weighed (designated as M_0_), and positioned within a tiny glass container.
(2) Vapour Generation: 1 g each of benzene, toluene, and xylene was introduced into a 100 mL sealed reagent container, permitting the liquids to volatilise and form a saturated vapour phase of BTX.
(3) Exposure Setup: The glass vial containing the membrane sample was positioned within the BTX-saturated bottle, guaranteeing that the membrane was solely exposed to the vapour phase, avoiding direct contact with the liquids.
(4) Adsorption Duration: The system was sustained at 30 °C for 24 h to facilitate adequate adsorption.
(5) Post-adsorption: The sample was extracted, and its ultimate weight (M₁) was documented.
(6) Calculation of Adsorption Capacity: The specific adsorption capacity (C, in mg/g) was determined using the subsequent equation, with each test conducted in triplicate:

C = (M_1_ – M_0_) × 1000 mg/g (1)

### 2.5. Regeneration of TMPP/P Membrane for Reuse

A cyclic adsorption–desorption experiment was undertaken to assess the reusability of TMPP/P membranes. Following the first BTX adsorption, the membranes underwent regeneration through thermal desorption by being placed in a convection oven at 65 °C under ambient pressure for 12 h to eliminate remaining adsorbates. The regenerated membranes were subsequently exposed to a fresh adsorption cycle under the same conditions. The procedure was reiterated for four successive cycles.

To guarantee the reliability of the data, each adsorption–desorption test was performed in triplicate. Subsequent to the fourth regeneration cycle, attenuated total reflectance-Fourier transform infrared spectroscopy (ATR-FTIR, Nicolet iS20, USA) was utilised to evaluate the structural integrity and chemical stability. Spectra were acquired within the region of 4000–400 cm^−1^ to compare the regenerated membrane with the original sample.

### 2.6. Adsorption Kinetics Models

To clarify the adsorption process and rate-controlling phases of BTX absorption by the electrospun TMPP/P membranes, three kinetic models were employed on the experimental data: the pseudo-first-order model, the pseudo-second-order model, and the Elovich model. These models elucidate the temporal adsorption dynamics.

The equations corresponding to each model are as follows:

(2) $$q_{t}=q_{e}(1-e^{-k_{1}t})$$

(3) $$\frac{t}{q_{t}}=\frac{1}{k_{2}q_{e}^{2}}+\frac{t}{q_{e}}$$

where *q_t_* is the adsorption capacity at time (mg/g); *q_e_* is the equilibrium adsorption capacity (mg/g); *k*_1_ is the pseudo-first-order rate constant (1/h); *k*_2_ is the pseudo-second-order rate constant (g/mg·h); *t* is the adsorption time (h); and the Elovich model can be described by the linear mathematical expression of Equation (4).

(4) $$q_{t}=\frac{1}{\beta }{ln}^{\left(\alpha \beta t+1\right)}$$

where *q_t_* is the adsorption capacity at time (mg/g); *α* is initial adsorption rate constant (mg/(g·h)); and *β* is surface coverage correlation constant (mg/g).

## 3. Results and Discussion

### 3.1. Characterisation of [P_(14)666_]TMPP and TMPP/P

The shape, thermal properties, crystallinity, and chemical structure of the PAN and TMPP/P membranes were thoroughly characterised.

**Figure 2:**
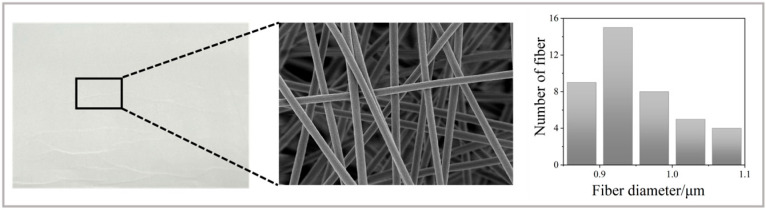
The physical image, SEM image, and particle size distribution diagram of PAN nanofiber membrane.

The electrospun PAN membrane had a consistent, smooth, and white fibrous structure. SEM analysis demonstrated that the fibres were randomly and densely interlaced, with diameters varying from 860 to 1080 nm and an average of 947 nm, thereby affirming the regularity and stability of the electrospinning process. Figure 2 depicts the SEM shape and diameter distribution of the unaltered PAN membrane.

**Figure 3:**
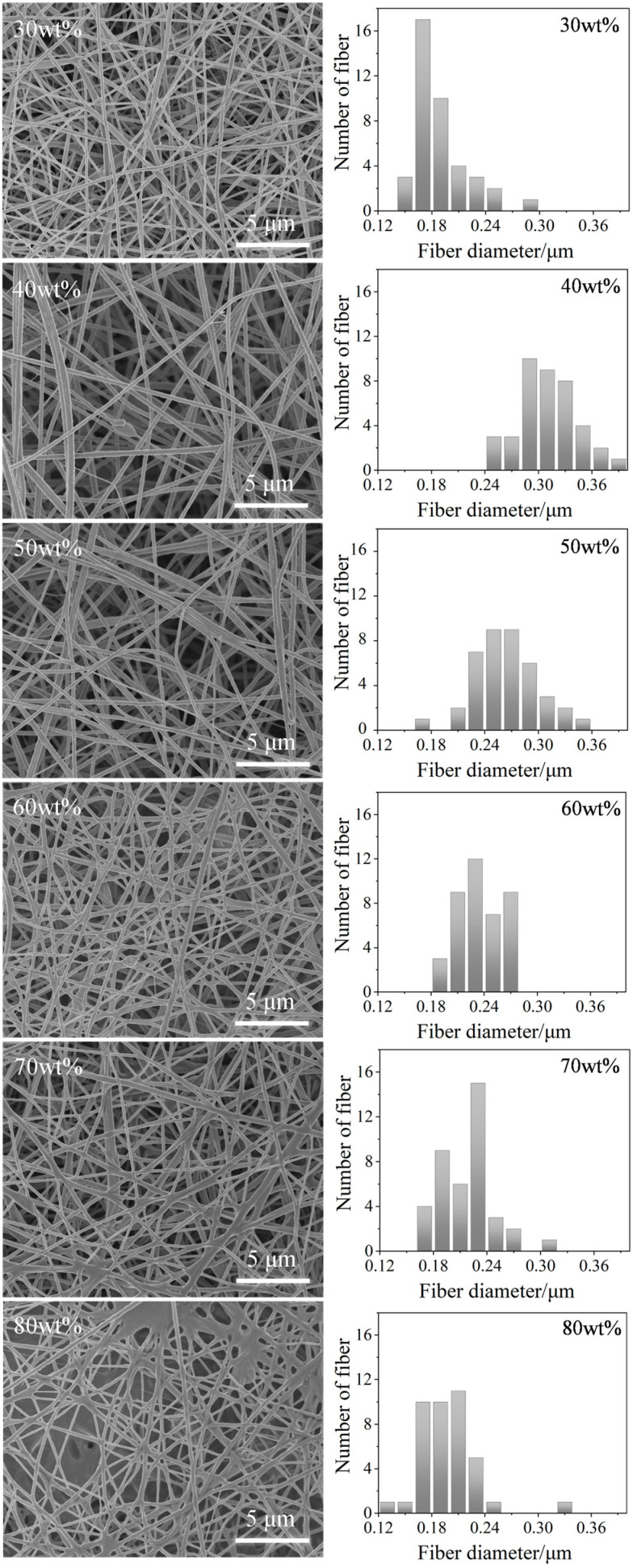
SEM image and particle size distribution diagram of TMPP/P.

Upon the incorporation of ionic liquid [P_(14)666_]TMPP into the PAN matrix, the resultant TMPP/P fibres exhibited a reduction in diameter and an enhancement in surface roughness. SEM pictures indicated that a higher IL content resulted in more pronounced fibre adherence, particularly in the 70 TMPP/P and 80 TMPP/P membranes, where flakes and fused structures were observed. The average fibre diameter first rose, reaching a maximum of 40 TMPP/P between 240 and 400 nm, before subsequently declining with additional IL loading, signifying a nonlinear effect of IL on fibre morphology. Figure 3 illustrates the structural evolution of TMPP/P membranes as the concentration of [P_(14)666_]TMPP increases.

Thermal stability was assessed using TGA in a nitrogen environment. Pure [P_(14)666_]TMPP exhibited a significant breakdown event within the temperature range of 238–396 °C. The weight reduction process for TMPP/P membranes was categorised into three distinct stages: (1) below 283 °C, associated with the evaporation of adsorbed water or residual solvent; (2) 283–360 °C, related to the cyclisation of PAN’s –C≡N groups; (3) 360–481 °C, encompassing the disintegration of the IL and subsequent pyrolysis/carbonization of PAN.

**Figure 4:**
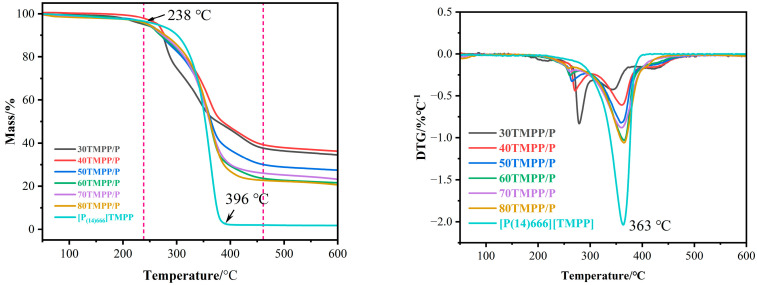
TG and DTG curves of TMPP/P and [P_(14)666_]TMPP.

The inclusion of IL altered the breakdown temperatures and enhanced the thermal stability of the composite membranes. Figure 4 illustrates the thermogravimetric analysis of PAN, IL, and composite membranes.

**Figure 5:**
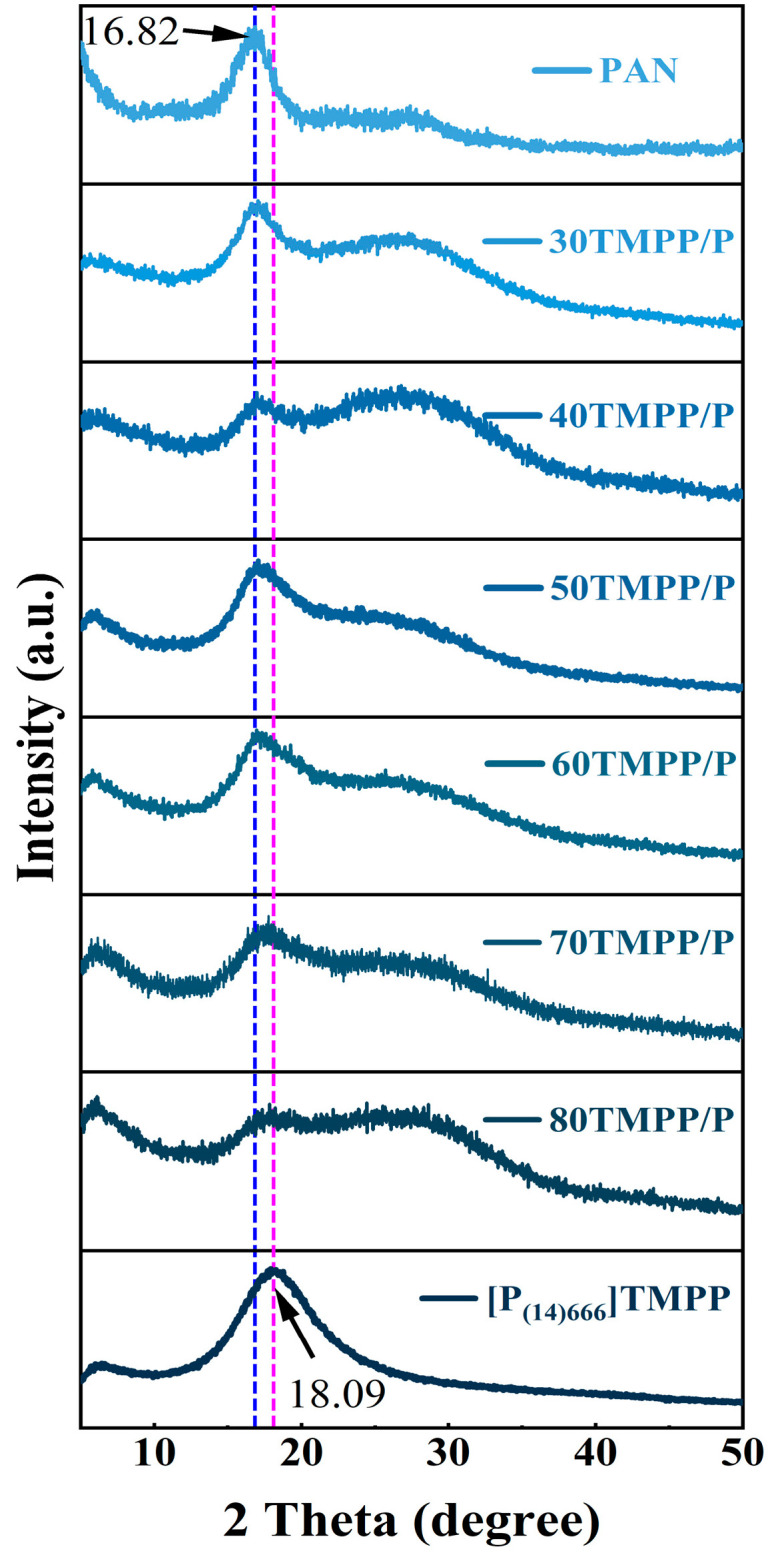
XRD patterns of PAN, TMPP/P and [P_(14)666_]TMPP.

XRD analysis additionally validated the structural modifications resulting from IL inclusion. The unblemished PAN membrane displayed a pronounced diffraction peak at 2θ = 16.8°, aligning with the (100) crystalline plane. The IL [P_(14)666_]TMPP exhibited a peak at 2θ = 18.09°. In the composite membranes, minimal ionic liquid (IL) content maintained the crystallinity of polyacrylonitrile (PAN), whereas elevated IL levels (≥70 wt%) diminished PAN peak intensity and amplified IL-related diffraction signals. This indicates that the IL impaired PAN crystallinity through physical mixing and partial molecular intercalation. Figure 5 illustrates the XRD signals that validate alterations in crystallinity.

**Figure 6:**
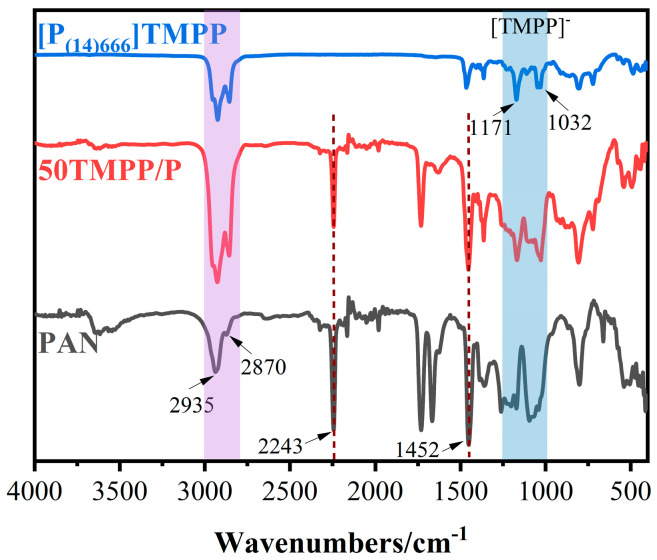
The FTIR spectra of PAN, 50 TMPP/P and [P_(14)666_]TMPP.

The infrared spectra of PAN, 50 TMPP/P, and [P_(14)666_][TMPP] are presented in Figure 6. The infrared spectrum of the PAN membrane exhibits distinct PAN features. The distinctive peak at 2243 cm^−1^ corresponds to the stretching vibration of the nitrile group (C≡N) in polyacrylonitrile, clearly indicating a characteristic peak of PAN. The peaks at 2935 cm^−1^ and 2870 cm^−1^ correspond to the asymmetric and symmetric stretching of aliphatic (C-H) CH_2_ groups, respectively, so further substantiating the PAN structure. The in-plane bending vibration of CH_2_ occurs at 1452 cm^−1^. The asymmetric and symmetric stretching vibrations of aliphatic C-H at 2935 cm^−1^ and 2870 cm^−1^ in the ionic liquid [P_(14)666_][TMPP] correspond to the C-H peak position of the PAN film. The absorption peak at 1452 cm^−1^ corresponds to the bending vibration of CH_2_, the peak at 1171 cm^−1^ represents the stretching vibration of P=O and the asymmetric stretching vibration of P-O-C in [TMPP]-phosphate, while the peak at 1032 cm^−1^ indicates the out-of-plane bending vibration of P-O-C in phosphate.

The aliphatic C-H of the ionic liquid in 50 TMPP/P and the C-H of PAN coincide at 2935 cm^−1^ and 2870 cm^−1^, but the absorption peaks of the residual PAN and the ionic liquid anion [TMPP]- are preserved in 50 TMPP/P without any displacement. The ionic liquid anion [TMPP]- did not establish a robust chemical connection with the C≡N of PAN. The study of XRD findings indicates that the combination of ionic liquid [P_(14)666_][TMPP] with PAN is a physical process.

### 3.2. VOC Absorption Performance of TMPP/P Membranes

**Figure 7:**
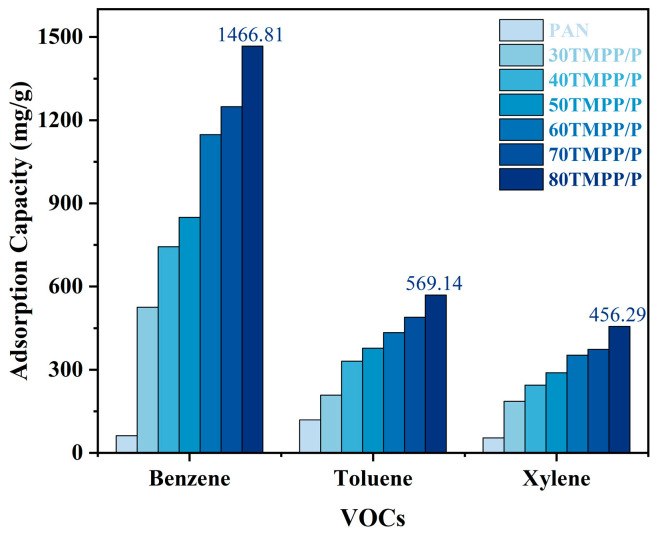
Adsorption capacity of BTX on PAN and TMPP/P.

Figure 7 illustrates the adsorption capabilities of PAN and TMPP/P membranes for benzene, toluene, and xylene (BTX). The electrospun PAN membrane functioned as a control, demonstrating moderate adsorption capabilities of 62.06 mg/g for benzene, 118.67 mg/g for toluene, and 54.11 mg/g for xylene. PAN exhibited the greatest affinity for toluene, presumably owing to the robust dipole–dipole interactions between the polar –C≡N groups in PAN and the comparatively polar structure of toluene.

Conversely, TMPP/P membranes exhibited markedly superior adsorption efficacy for all three VOCs. A discernible pattern was noted indicating that adsorption capacity augmented with the ionic liquid concentration. The 80 TMPP/P membrane attained maximum adsorption capacities of 1466.81 mg/g for benzene, 569.14 mg/g for toluene, and 456.29 mg/g for xylene, representing enhancements of 23.6-, 4.8-, and 8.4-fold, respectively, compared to the unmodified PAN membrane.

This improvement can be ascribed to the particular chemical interactions between the quaternary phosphonium ionic liquid [P_(14)666_]TMPP and aromatic VOCs. The primary adsorption mechanisms are hydrogen bonding, π–π stacking, and C–H···π interactions. The acidic α-hydrogens of the phosphonium cation can rapidly establish C–H···π interactions with the delocalised π-electrons of the benzene ring due to benzene’s non-polarity and the shortest molecular size among the three. This results in the maximum adsorption [28,29,30,31].

In the instances of toluene and xylene, further hydrogen bonding interactions may transpire between the ionic liquid anion and the methyl groups on the aromatic rings. Nonetheless, C–H···π interactions constitute the principal adsorption force for all three VOCs. These results correspond with previously documented processes for VOC collection utilising phosphonium-based ionic liquids [30,31].

### 3.3. Regeneration of TMPP/P Membranes for Reuse

**Figure 8:**
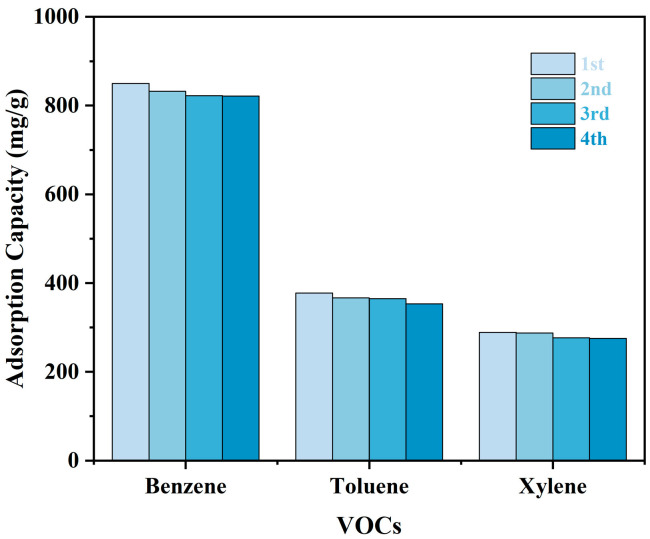
Recycling adsorption capacity variation of 50 TMPP/P.

The reusability of the TMPP/P membranes was assessed by four successive adsorption–desorption cycles, utilising the 50 TMPP/P membrane as a typical specimen. Figure 8 illustrates that the adsorption capabilities for benzene, toluene, and xylene showed minimal reductions following four regeneration cycles. The capacity losses were 3.2%, 6.6%, and 4.8%, respectively, exemplifying exceptional operating stability and regeneration performance.

**Figure 9:**
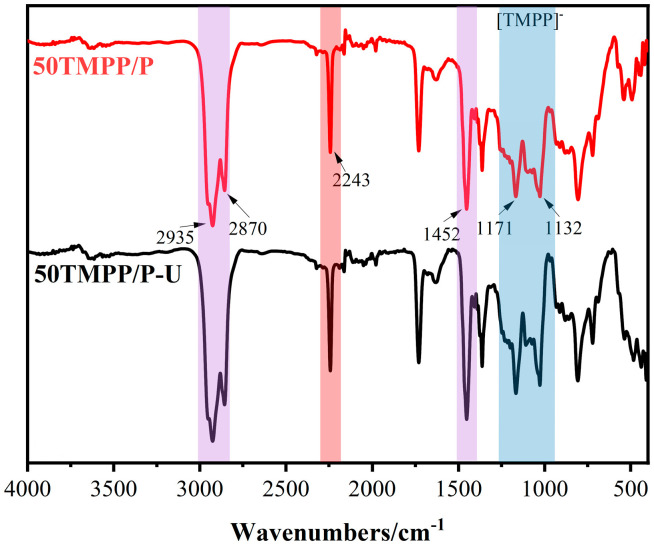
The FTIR spectra of regenerated 50 TMPP/P.

ATR-FTIR research was conducted on the regenerated 50 TMPP/P membrane to examine any structural or chemical alterations post-regeneration. Figure 9 illustrates that the IR spectra of the regenerated membrane closely resemble those of the virgin membrane, exhibiting no discernible changes, the loss of distinctive peaks, or the introduction of new peaks. The retained vibrational bands verify that the functional groups of both PAN and [P_(14)666_]TMPP were unaltered during the thermal desorption procedure.

The combined data indicate that the adsorption of BTX onto TMPP/P membranes is predominantly influenced by physical interactions (e.g., C–H···π bonding), which do not modify the chemical structure of the membrane. As a result, the membranes may be restored and utilised again with negligible performance degradation, rendering them a viable and sustainable choice for VOC removal applications.

### 3.4. Kinetic Models Fitting

**Figure 10:**
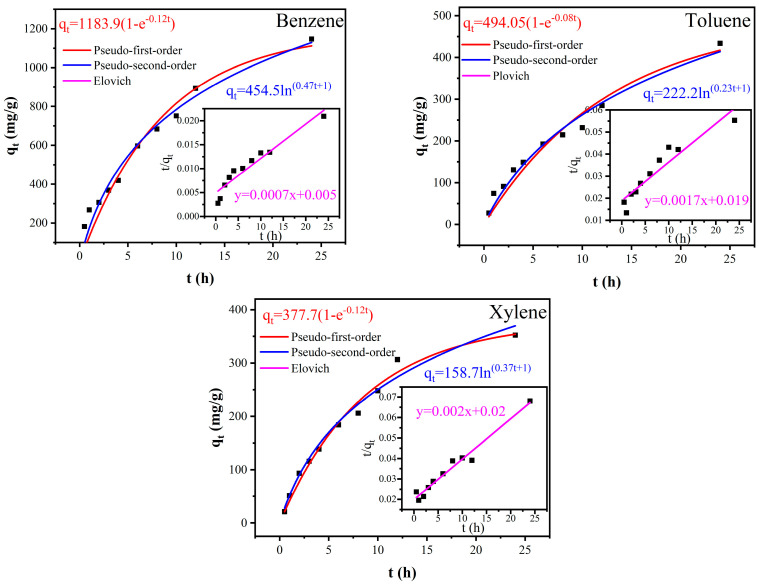
The kinetics model fitting curve of TMPP/P membrane adsorbing BTX.

To examine the adsorption characteristics of BTX on TMPP/P membranes, nonlinear kinetic fitting was performed with Origin 2021 software, employing three widely recognised models: the pseudo-first-order, pseudo-second-order, and Elovich models. The kinetic parameters obtained from the fitting findings are presented in Table 2, and the nonlinear fitting curves are illustrated in Figure 10. The adsorption of BTX on TMPP/P membranes was accurately characterised by both the pseudo-first-order and Elovich models, as indicated by the high correlation coefficients (R^2^). Of the three models, the Elovich model had the most superior fit, suggesting that the adsorption process is predominantly influenced by heterogeneous surface diffusion and chemisorption properties.

In the case of xylene, the pseudo-second-order model produced a high R^2^ value of 0.97, indicating that xylene adsorption may entail a rate-limiting chemisorption step, likely attributable to its greater molecular size and reduced volatility relative to benzene and toluene. The results indicate that the adsorption kinetics of BTX onto TMPP/P membranes are predominantly influenced by a mixture of surface contact processes, with various VOC species displaying minor differences in rate-controlling steps based on their molecular characteristics.

### 3.5. Comparison of the Adsorption Performance of TMPP/P Membranes with Other Adsorbents

This work assessed the competitiveness of the TMPP/P membranes by comparing their adsorption capacities for benzene, toluene, and xylene with those of representative adsorbents documented in the literature, as outlined in Table 3.

The findings indicate that the 80 TMPP/P membrane displays markedly superior adsorption capabilities for all three VOCs. For instance, its absorption capacity for benzene (1466.81 mg/g) significantly exceeds the values documented for conventional materials like activated carbon, zeolites, and MOF-based composites. The adsorption capabilities for toluene and xylene surpass those of conventional polymer-based and ionic liquid-immobilised membranes.

The improved performance is due to the synergistic effects of the electrospun nanofiber structure, which offers high surface area and porosity, and the strong affinity of the quaternary phosphonium ionic liquid for aromatic hydrocarbons via C–H···π interactions and advantageous dispersion forces. These results validate that the TMPP/P membrane is not only an exceptionally effective adsorbent for BTX but also a viable contender for practical VOC removal applications in air purification systems.

## 4. Conclusions

This study effectively manufactured a series of TMPP/P composite nanofiber membranes using electrospinning. The ionic liquid was physically incorporated into the PAN matrix, yielding membranes with higher thermal stability and markedly improved adsorption capabilities for volatile aromatic chemicals.

Of the synthesised membranes, the 80 TMPP/P sample had the most refined fibre morphology and superior adsorption efficacy, attaining capacities of 1466.81 mg/g for benzene, 569.14 mg/g for toluene, and 456.29 mg/g for xylene. The enhanced absorption is ascribed to robust C–H···π interactions between the cations of the ionic liquid and aromatic volatile organic compounds.

Thermal desorption tests verified that the TMPP/P membranes preserved their structural integrity and adsorption efficacy after four regeneration cycles, indicating exceptional reusability. The integration of elevated adsorption efficiency, excellent thermal stability, and reusability underscores the promise of these electrospun ionic liquid membranes as high-performance adsorbents for the removal of volatile organic compounds in air purification applications.

This study presents a pragmatic and scalable approach for incorporating ionic liquids into nanofiber membrane systems and delivers significant insights for the development of advanced functional adsorption materials. Future research may involve the further integration of imidazolium, ammonium, and pyridinium-based ionic liquids into nanofiber membranes through electrospinning technology to examine the impact of various ionic liquid composite membranes on the performance of VOC adsorption.

## Figures and Tables

**Table 1:** The [P_(14)666_]TMPP ratios in electrospinning solution and [P_(14)666_]TMPP/PAN membranes.

IL/PAN Name Abbreviation	Content of PAN (g)	Content of IL (g)	IL Ratio in Electrospinning Solution (wt%)
PAN	1.0	0	0
30 TMPP/P	0.7	0.3	30
40 TMPP/P	0.6	0.4	40
50 TMPP/P	0.5	0.5	50
60 TMPP/P	0.4	0.6	60
70 TMPP/P	0.3	0.7	70
80 TMPP/P	0.2	0.8	80

**Table 2:** Kinetics model parameters.

Kinetic Models	Benzene	Toluene	Xylene
Pseudo-first-order kinetics model			
q_e_	1183.94	494.07	377.70
k_1_	0.11679	0.0776	0.11509
R^2^	0.95009	0.96036	0.98555
Pseudo-second-order kinetics model			
q_e_	1394.16	574.71	505.05
k_2_	0.0001039	0.00016463	0.00019691
R^2^	0.92471	0.89581	0.97145
Elovich model			
α	212.18	50.10	59.29
β	0.0022	0.0045	0.0063
R^2^	0.9721	0.9772	0.9926

**Table 3:** The comparison of adsorption capacities of TMPP/P membrane and some other adsorption materials for BTX.

Adsorption Materials	Adsorbate	Adsorption Capacity (mg/g)	References
PDMS/AC-150	Benzene	360	(Liu et al. [32])
PDMS/AC-250	Benzene	350	(Liu et al. [32])
AC	Benzene	336	(Liu et al. [32])
OPASS/UiO-66-NH_2_	Benzene	20.3	(Wei et al. [27])
80 TMPP/P membrane	Benzene	1466.8	In current study
AC	Toluene	364.96	(Liu et al. [32])
OPASS/UiO-66-NH_2_	Toluene	28.7	(Wei et al. [27])
[Emim]BF_4_	Toluene	4.5	(Ma et al. [33])
AC	Toluene	512.03	(Orhan et al. [34])
80 TMPP/P	Toluene	569.1	In current study
[Emim]BF_4_	Xylene	5.5	(Ma et al. [33])
80 TMPP/P	Xylene	456.3	In current study

## Data Availability

The data will be made available upon request.
